# Big Data analysis on autopilot?

**DOI:** 10.1186/1756-0381-6-22

**Published:** 2013-12-06

**Authors:** Scott M Williams, Jason H Moore

**Affiliations:** 1 Department of Genetics and Institute for Quantitative Biomedical Sciences, The Geisel School of Medicine, Dartmouth College, One Medical Center Dr., Lebanon, Lebanon, NH 03756, USA

## 

Biomedical sciences, especially fields such as genomics, are becoming Big Data fields, driven, to a large extent, simply by the ability to generate enormous data sets. For fields such as biology, where data has traditionally been small, the influx of Big Data, noise and all, has caused a need to rapidly shift research practices. Ways of dealing with these data have led to importing collaborators from statistics, computer science, and physics. However, these new “biologists” are simply not biologists and classically trained biologists are not “Big Data researchers”. The confluence of these fields unfortunately has not led to the improvement of biological understanding via what should be synergy, but to a completely unfortunate shifted landscape of research. The new landscape involves Big Data use that reductionist biologists can comprehend quickly. Simply put, it has become the use of Big Data with a reductionist twist, or performing mostly reductionist analyses of Big Data. Of course, reductionism has been extremely fruitful in unraveling mechanisms for many key biological processes, but the translation of these processes into understanding of complex phenotypes, such as common human disease, has been less simple.

So instead of cleverly and creatively using Big Data to filter noise from signal, and to understand inherently complex biological problems, the world of Big Data biology has effectively taken the lowest common denominator between Big Data and biology. In other words analyses have been reduced to simple outputs that can be readily interpreted using traditional mindsets. We have reduced the use of Big Data to analyses and interpretation that is often the equivalent of being on autopilot.

Autopilot, especially of large commercial planes, has many advantages for normal flight, making the pilot’s job easier. However, as recently seen there are limits to its pervasive implementation – not the least of which is the distancing of the pilot from the real control and understanding of flight. Under normal, uncomplicated flight, this is not an issue, but when emergencies or unusual difficulties occur the dependence on autopilot has the unintended consequence of diminishing pilot reaction to complex flight conditions. Such problems arise under conditions such as those of Air France flight 447 over the Atlantic, when the plane’s autopilot “suddenly disengaged and a stall warning activated. The senior co-pilot then said: “What’s happening? I don’t know, I don’t know what’s happening [1]”. In reaction, the co-pilot pulled the nose up – exactly opposite to what should be done. The pilot responded, but too late, and the plane crashed into the ocean killing all 228 people onboard. Simply, the co-pilot was lulled into complacency by dependence on an automated process.

In Big Data biology we may have been similarly lulled by the plug and play analytical programs that are generally applied in the literature. Instead, the real issue should be: what is useful data and how do we extract it from the haystack of large-scale data? As a field, biology has too often developed and embraced standard analytical methods and presentations that do not attempt to understand complexity, but instead remove uniqueness of a given trait where it needs to remain. These approaches have resulted in too much science on autopilot, where the scientists only touch the controls at the last minute or upon landing of the study, and machines do all of the rest. This has often resulted in our ability to use Big Data to rediscover truths known for decades when investigators had to be creatively engaged in the collection, analyses and thoughtful interpretation of data.

This is not to say that having lots of data and very smart people around is bad, it only means that we need to refocus how we approach the Big Data that will certainly be collected, using clever analyses and synergy between those who can analyze it this way and those who understand the phenotypes and biological processes that these data represent. *BioData Mining* can serve as a vortex for this kind of research and we hope to engage diverse cohorts of researchers to do so.
